# Identification and immunological characterization of endoplasmic reticulum stress-related molecular subtypes in bronchopulmonary dysplasia based on machine learning

**DOI:** 10.3389/fphys.2022.1084650

**Published:** 2023-01-09

**Authors:** Ziyu Tao, Yan Mao, Yifang Hu, Xinfang Tang, Jimei Wang, Ni Zeng, Yunlei Bao, Fei Luo, Chuyan Wu, Feng Jiang

**Affiliations:** ^1^ Department of Ultrasound, Obstetrics and Gynecology Hospital of Fudan University, Shanghai, China; ^2^ Department of Pediatrics, The First Affiliated Hospital of Nanjing Medical University, Nanjing, China; ^3^ Department of Geriatric Endocrinology, The First Affiliated Hospital of Nanjing Medical University, Nanjing, China; ^4^ Department of Nephrology, The Affiliated Lianyungang Oriental Hospital of Xuzhou Medical University, The Affiliated Lianyungang Oriental Hospital of Kangda College of Nanjing Medical University, The Affiliated Lianyungang Oriental Hospital of Bengbu Medical College, Lianyungang, China; ^5^ Department of Neonatology, Obstetrics and Gynecology Hospital of Fudan University, Shanghai, China; ^6^ Department of Dermatology, Affiliated Hospital of Zunyi Medical University, Zunyi, China; ^7^ Department of Rehabilitation Medicine, The First Affiliated Hospital of Nanjing Medical University, Nanjing, China

**Keywords:** bronchopulmonary dysplasia, machine learning, endoplasmic reticulum stress, immune infiltration, prediction model

## Abstract

**Introduction:** Bronchopulmonary dysplasia (BPD) is a life-threatening lung illness that affects premature infants and has a high incidence and mortality. Using interpretable machine learning, we aimed to investigate the involvement of endoplasmic reticulum (ER) stress-related genes (ERSGs) in BPD patients.

**Methods:** We evaluated the expression profiles of endoplasmic reticulum stress-related genes and immune features in bronchopulmonary dysplasia using the GSE32472 dataset. The endoplasmic reticulum stress-related gene-based molecular clusters and associated immune cell infiltration were studied using 62 bronchopulmonary dysplasia samples. Cluster-specific differentially expressed genes (DEGs) were identified utilizing the WGCNA technique. The optimum machine model was applied after comparing its performance with that of the generalized linear model, the extreme Gradient Boosting, the support vector machine (SVM) model, and the random forest model. Validation of the prediction efficiency was done by the use of a calibration curve, nomogram, decision curve analysis, and an external data set.

**Results:** The bronchopulmonary dysplasia samples were compared to the control samples, and the dysregulated endoplasmic reticulum stress-related genes and activated immunological responses were analyzed. In bronchopulmonary dysplasia, two distinct molecular clusters associated with endoplasmic reticulum stress were identified. The analysis of immune cell infiltration indicated a considerable difference in levels of immunity between the various clusters. As measured by residual and root mean square error, as well as the area under the curve, the support vector machine machine model showed the greatest discriminative capacity. In the end, an support vector machine model integrating five genes was developed, and its performance was shown to be excellent on an external validation dataset. The effectiveness in predicting bronchopulmonary dysplasia subtypes was further established by decision curves, calibration curves, and nomogram analyses.

**Conclusion:** We developed a potential prediction model to assess the risk of endoplasmic reticulum stress subtypes and the clinical outcomes of bronchopulmonary dysplasia patients, and our work comprehensively revealed the complex association between endoplasmic reticulum stress and bronchopulmonary dysplasia.

## Introduction

Bronchopulmonary dysplasia (BPD) is a pulmonary condition that affects preterm newborns and is hallmarked by suppressed lung development and lung damage, requiring life-saving medical interventions like mechanical ventilation and oxygen treatment (Thebaud et al., 2019). Consequently, it is one of the conditions that is considered to be the most life-threatening in neonatal intensive care units (Bancalari and Jain, 2019). Even though there have been tremendous breakthroughs in maternal and neonatal care, the prevalence of BPD in preterm babies remains between 20% and 40% (Sillers et al., 2020). BPD may result in impaired lung development and permanent impairment (Gilfillan et al., 2021). BPD may also cause harm to the neurological system and other systemic organs (Shukla and Ambalavanan, 2021). However, the pathogenesis of BPD is poorly known, and there are few early diagnostic tests and precise prevention or therapy approaches for this disease.

The endoplasmic reticulum (ER) is essential for maintaining the intracellular environment’s equilibrium (Zeeshan et al., 2016). Under persistent stress, the disturbance of ER homeostasis may result in ER stress, as demonstrated by alterations in the Ca^2+^ levels in the cells and the overaccumulation of misfolded or unfolded proteins, which ultimately leads to numerous protein-folding diseases, including BPD (Adamopoulos et al., 2014; Leprivier et al., 2015). It is generally believed that prematurely born infants display oxidative stress shortly after delivery owing to their immature antioxidant defenses and the fast increase in oxygen tension caused by medical intervention (Bonadies et al., 2020). Numerous adaptive survival mechanisms are triggered in response to a sudden rise in oxidative stress, such as the UPR (a reaction to variations in chaperone function), resulting in increased ER stress and reduced protein production to facilitate the restoration of proteostasis (Hu et al., 2020; Kepp and Galluzzi, 2020). Both ER stress and oxidative stress are two connected conditions associated with a variety of lung diseases (Marciniak, 2017; Chen et al., 2018). Recent research indicates that ER stress is elevated in the lungs of numerous recognized BPD animal models (Pritchard et al., 2022). These investigations showed that ER stress may be an effective therapeutic target for BPD. However, the particular molecular processes of ER in controlling the course of BPD have yet to be completely explained and need more research.

We investigated the expression patterns of ER stress-related genes (ERSGs) between BPD and control samples, as well as the association between differentially expressed genes (DEGs), linked to ER stress immune features. Then, we grouped BPD patients into two ER stress-related clusters and analyzed the differences (variations) in immune cell infiltration between the two groups. The WGCNA approach was then used to detect cluster-specific DEGs, and enriched bioactivities and pathways were determined based on these cluster-specific DEGs. Moreover, the analysis of multiple machine-learning approaches resulted in the development of a prediction model for identifying patients who have distinct molecular clusters After that, we verified the nomogram, calibration curve, decision curve analysis (DCA), and the predictive model’s performance, which offered some additional unique knowledge for predicting BPD clusters and relevant risk.

## Materials and methods

### Acquisition and preprocessing of data

Gene Expression Omnibus (GEO) was searched to acquire two microarray datasets (GSE32472 and GSE108756) associated with BPD. Microarray profiles of gene expression in neonates with BPD were reported by GSE32472 using blood samples taken around the 5th, 14th, and 28th days of life. For reliability, we chose 100 blood samples on around the 28th day, when a more precise diagnosis of BPD could be established, which mainly concluded 38 controls and 62 patients with BPD. GSE108756 included six control samples and five BPD samples and was used as an external validation set. To standardize the expression data, the quantile normalization function in the limma package of R was used. The ERSGs were obtained from two gene sets (GOBP response to endoplasmic reticulum stress and GOBP regulation of response to endoplasmic reticulum stress) of MSigDB.v2022 and 260 ERSGs were collected (Supplementary Table S1).

### Analysis of immune cell infiltration

To calculate the relative abundance of 22 different types of immune cells in each sample using the processed gene expression profiles, the CIBERSORT method and LM22 signature matrix were used (Newman et al., 2015). When calculating the inverse fold product probability value, CIBERSORT employs Monte Carlo sampling. We only counted immune cell fractions with *p*-values below .05 as accurate. The sum of the 22 immune cell proportions in each sample was 1.

### Analysis of the relationship between ERSGs and infiltrating immune cells

We examined correlation coefficients between ERSGs expression and the relative proportion of immune cells as a further demonstration of the connection between ERSGs and BPD-associated immune features. *p*-values < .05 for the spearman correlation coefficient indicated a statistically significant relationship. Lastly, the “correplot” R package was used to visually display the findings.

### Unsupervised clustering of BPD patients

We analyzed the differences in ERSGs between BPD and control groups, and the screening criteria were |logFC| > 0 and *p* < .001 for genes with significant differences. We got 31 differentially expressed ERSGs. Using the expression patterns of 31 ERSGs as input, we executed an unsupervised clustering analysis utilizing the “ConsensusClusterPlus” R package (Wilkerson and Hayes, 2010), which used the k-means method and 1,000 interactions to classify the 62 BPD samples into distinct clusters. On the basis of the consensus matrix, the cumulative distribution function (CDF) curve (Boso et al., 2014), and the consistent cluster score (>.9), we determined that *k* = 2 was the maximum number of subtypes in our analysis and thoroughly evaluated the optimal cluster number.

### Gene set variation analysis (GSVA)

The “GSVA” package (Hanzelmann et al., 2013) in R was employed to perform enrichment analysis, which helped to reveal the variations in enriched gene sets across various ER stress clusters. From the MSigDB website database, we obtained the “c2.cp.kegg.symbols.gmt” and “c5.go.symbols.gmt” files to conduct additional GSVA analysis. By comparing GSVA scores across several ER stress clusters, the “limma” R program was used to determine the pathways and biological functions that were expressed differently. Outcomes in the GSVA score with a t-value greater than two were judged significant.

### Weighted gene co-expression network analysis (WGCNA)

The “WGCNA” package in R was employed to execute WGCNA and find co-expression modules (Langfelder and Horvath, 2008). To ensure the reliability of high-quality findings, the top 25% of genes with the greatest variation were subjected to WGCNA analyses. The optimal soft thresholding power was used to generate the weighted adjacency matrix, which was then used as the basis for a topological overlap matrix (TOM). Additionally, the TOM dissimilarity measure (1-TOM) predicated on the hierarchical clustering tree technique was used to create modules with a minimum module size of 100. A different color was chosen at random for each module. The global gene expression patterns in each module were denoted by the eigengene of the corresponding module. Modular significance (MS) demonstrated the connection between modules and illness conditions. The link between a gene and its clinical phenotypes was termed gene significance (GS).

### The development of a prediction model using various machine-learning techniques

We used the “caret” R package to develop machine learning models, which include an eXtreme Gradient Boosting (XGB), generalized linear model (GLM), support vector machine model (SVM), and random forest model (RF), premised on two distinct ERSG clusters. RF is a machine learning ensemble method that predicts categorization or regression using several, unrelated decision trees (Rigatti, 2017). The SVM technique allows for the generation of a hyperplane in the characteristic space that has a hyperplane that maximizes the margin to discriminate between negative and positive examples (Giraldo et al., 2006). GLM is a variant of the linear regression technique used to examine the link between normal distributions of dependent data and continuous or categorical independent characteristics. XGB is a gradient-boosting-based ensemble of boosted trees that can quantitatively compare model complexity and classification error (Lee and Nelder, 2002). Cluster-specific DEGs were chosen as explanatory variables, whereas the response variables were distinct clusters. We classified 62 BPD samples at random into a training set (consisting of 70%) and a validation set (consisting of 30%). Machine learning models were run with default settings and evaluated by means of 5-fold cross-validation, and parameter optimization was done automatically by the caret package using grid search. To clearly explain the link between these four machine learning models and to compare their residual distributions and feature significance, the “DALEX” package was implemented. To display the area under ROC curves, the “pROC” (Robin et al., 2011) R program was used. As a result, the best machine learning model was selected, and the top five factors served as the key predictor genes for BPD.

### Development and verification of a nomogram model

With the aid of the “rms” R package, we developed a nomogram to analyze BPD cluster-related occurrence. There is a corresponding score assigned to each predictor, and this value is added together to get the “total score.” To evaluate the nomogram model’s prediction ability, we used the DCA (Vickers and Elkin, 2006) and calibration curve.

### Independent validation analysis

The ROC analyses were used to verify the predictive model’s capacity to differentiate between BPD and non-BPD controls using the data set GSE108756. The “pROC” R package was used to visualize ROC curves.

## Results

### Dysregulation of ERSGs

**FIGURE 1:**
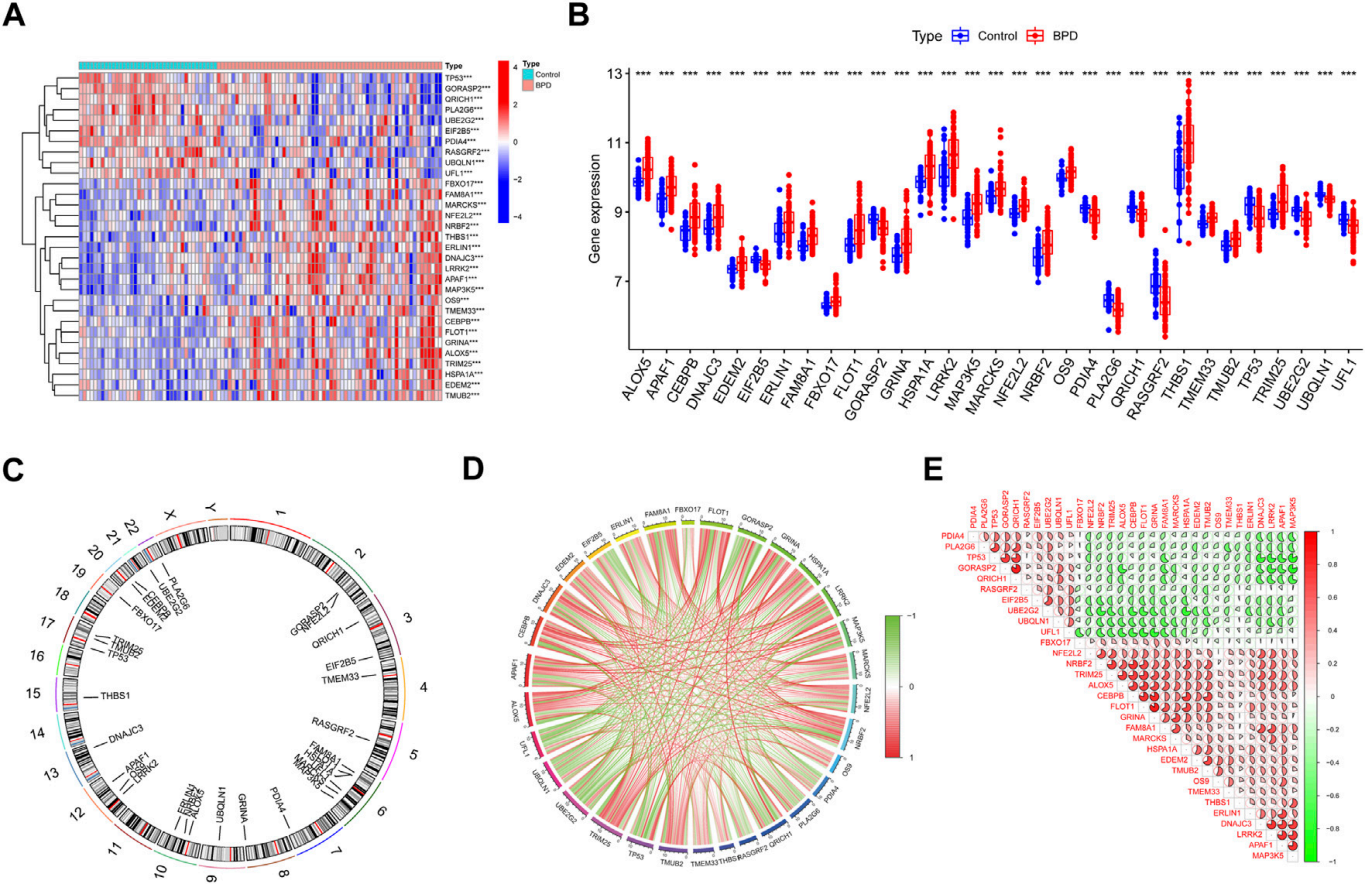
Identification of dysregulated ERSGs in BPD. **(A)** DE-ERSGs were presented in the heatmap. **(B)** Boxplots showed the expression of DE-ERSGs between BPD and control samples. ***p < .001. **(C)** The location of DE-ERSGs on chromosomes. **(D)** Gene relationship network diagram of DE-ERSGs. **(E)** Correlation analysis of DE-ERSGs.

We initially extensively made a comparison of the expression patterns of 260 ERSGs across BPD and control samples based on the GSE32472 dataset, to elucidate the biological involvement of ERSGs in the onset and advancement of BPD. In total, 31 ERSGs were determined as the differentially expressed ERSGs (DE-ERSGs) according to the criteria of |logFC| > 0 and *p* < .001 (Figures 1A–C). To further probe whether ERSGs played a crucial function in the onset and advancement of BPD, we conducted a correlation study across these DE-ERSGs. Many of the ERSGs presented strong synergistic effects (Figure 1D). Additional evidence of the remarkable link between these DE-ERSGs was provided by the gene interaction network diagram (Figure 1E).

### Activation of the immune responses in BPD patients

**FIGURE 2:**
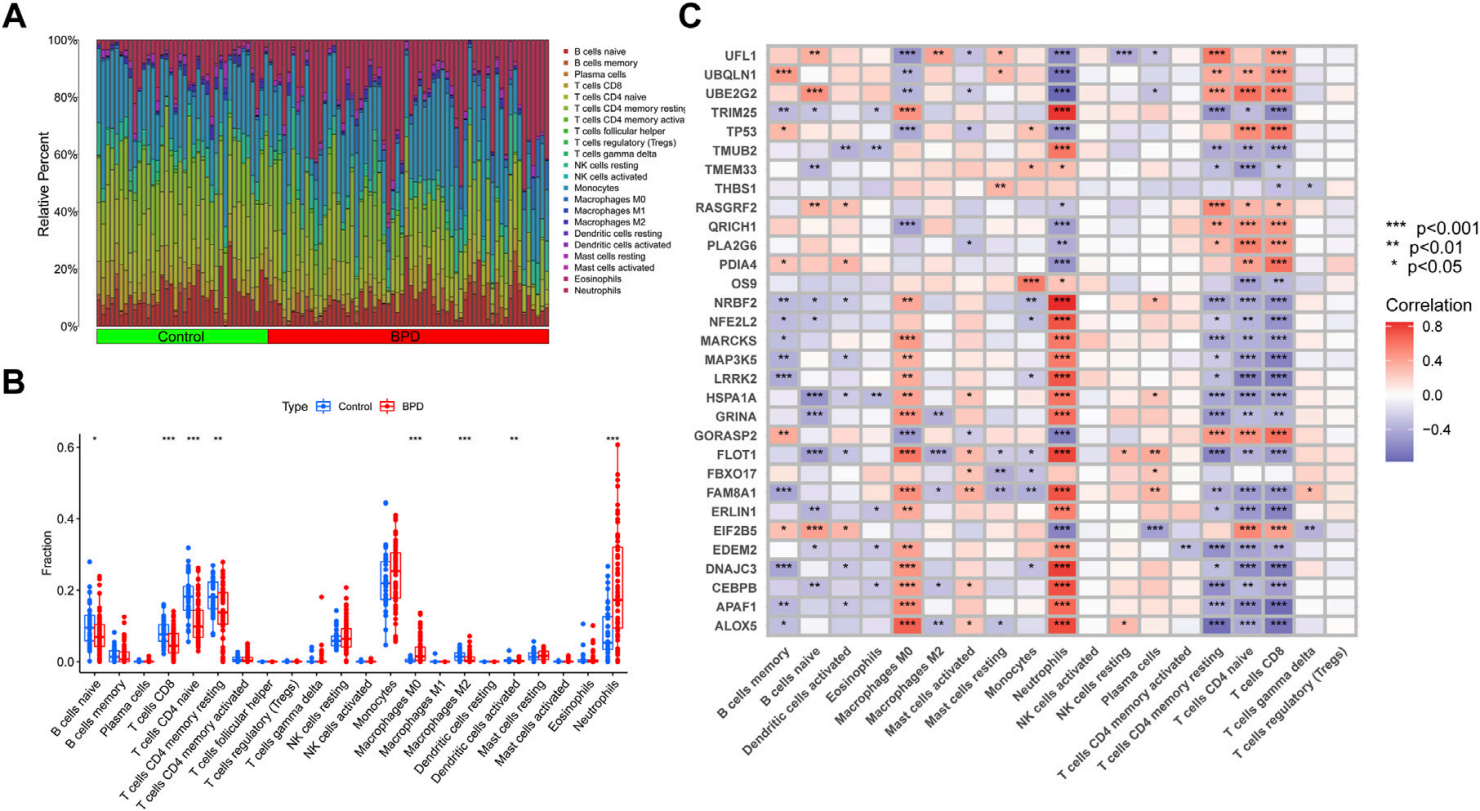
Identification of molecular and immune characteristics between BPD and control samples. **(A)** The relative abundances of 22 infiltrated immune cells between BPD and control samples. **(B)** Boxplots showed the differences in immune infiltrating between BPD and control samples. **(C)** Correlation analysis between DE-ERSGs and infiltrating immune cells. *p < .05, **p < .01, ***p < .001.

Immune cell infiltration analysis using the CIBERSORT method demonstrated a difference in the percentages of 22 types of infiltrated immune cells between BPD and control samples, providing more evidence for the existence of immune system differences between the two groups (Figure 2A). The findings illustrated that BPD patients exhibited a greater infiltration level of monocytes, macrophages M0, neutrophils, naive CD4^+^ T cell, and lower levels of dendritic cells activated, macrophages M2, resting memory CD4 T cells, and CD8 T cells (Figure 2B), pointing to immune system changes as a potential root cause of BPD. Nevertheless, a study of correlations found strong associations between ERSGs and neutrophils, macrophages, resting CD4 memory T cells, naïve CD4 T cells, and CD8 T cells (Figure 2C). From these findings, ERSGs could perform a crucial function in controlling the molecular and immunological infiltration status of BPD patients.

### Determination of ERSGs clusters in BPD

**FIGURE 3:**
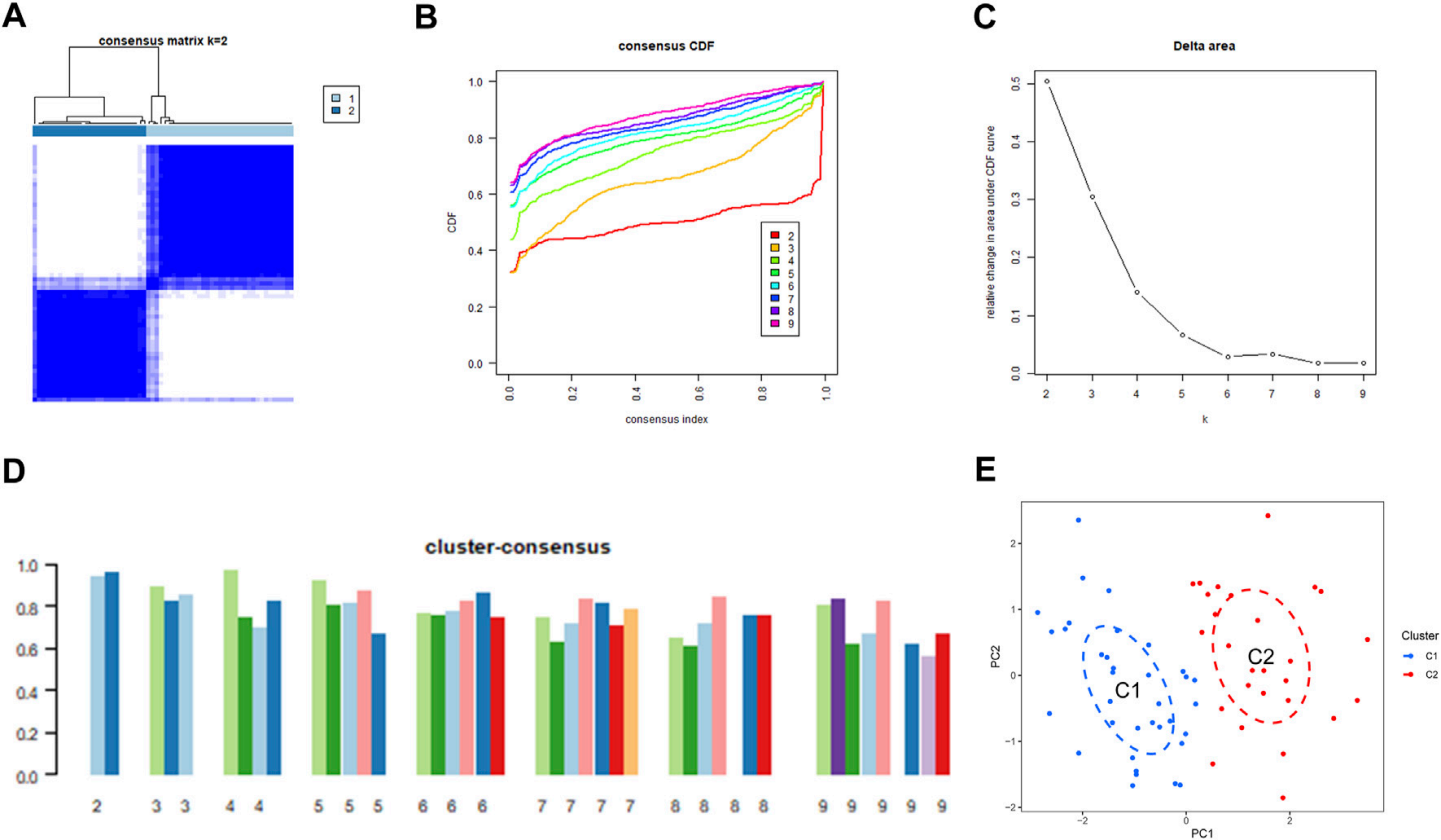
Identification of ER stress-related molecular clusters in BPD. **(A)** Consensus clustering matrix when *k* = 2. **(B–D)** Representative CDF curves. **(E)** PCA visualizes the distribution of two subtypes.

We used a consensus clustering technique to classify the 62 samples according to the expression patterns of 31 DE-ERSGs, thus elucidating the ER stress-associated expression profiles in BPD. Cluster sizes were most consistent with a *k* value of 2 (*k* = 2), whereas CDF curves showed little variation between consensus indices of .2 and .8 (Figures 3A–C). Additionally, only when *k* = 2, each subtype’s consistency score was >.9 (Figure 3D). Hence, two clusters were finally identified as optimal. Further principal component analysis (PCA) (Ben and Ben, 2021) findings showed a remarkable variation between the aforementioned clusters (Figure 3E).

### Differentiation of ERSGs and immune infiltration features between ERSGs clusters

**FIGURE 4:**
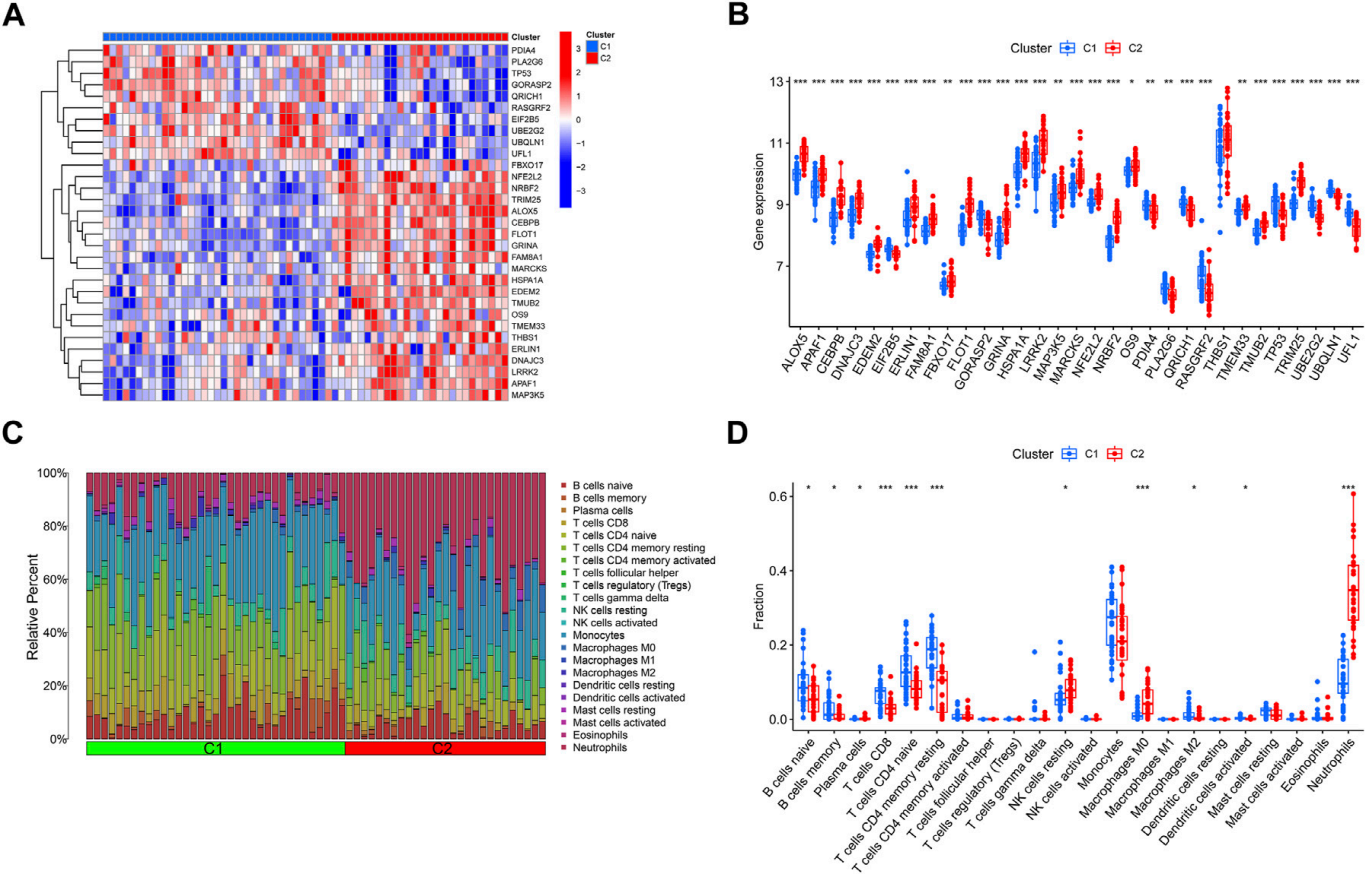
Identification of molecular and immune characteristics between the two ER stress clusters. **(A)** Expression patterns of DE-ERSGs between two ER stress clusters were presented in the heatmap. **(B)** Boxplots showed the expression of DE-ERSGs between two ER stress clusters. **(C)** The relative abundances of 22 infiltrated immune cells between two ER stress clusters. **(D)** Boxplots showed the differences in immune infiltrating between two ER stress clusters. *p < .05, **p < .01, ***p < .001.

The expression variations of ERSGs between clusters 1 and 2 were first thoroughly evaluated, allowing for the investigation of the molecular variations across the two categories. Two different ERS patterns were found, each with its unique expression landscape of ERSGs (Figures 4A, B). Furthermore, immune infiltration study findings revealed a changed immune milieu between ER stress clusters 1 and 2 (Figure 4C). Cluster two exhibited higher proportions of macrophages M0, neutrophils, and NK cells resting, whereas the levels of B cells naive, B cells memory, T cells CD8, T cells CD4 memory resting, and monocytes were relatively greater in cluster 1 (Figure 4D). These results confirmed that the two clusters based on the ERSGs had different immune environments, which provided a strong case for foundational genetic changes that link ERS, immune cell infiltration, and the remodeling seen with BPD.

### Screening of gene modules and creation of co-expression networks

**FIGURE 5:**
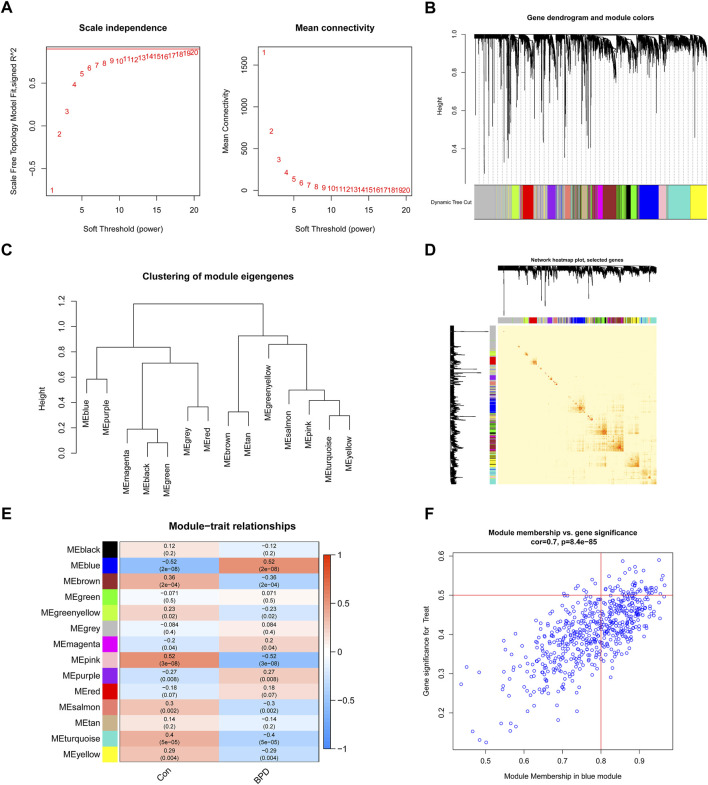
Co-expression network of DEGs in BPD. **(A)** The selection of soft threshold power. **(B)** Cluster tree dendrogram of co-expression modules. **(C)** Representative of clustering of module eigengenes. **(D)** Representative heatmap of the correlations among 14 modules. **(E)** Correlation analysis between module eigengenes and clinical status. **(F)** Scatter plot between module membership in the blue module and the gene significance for BPD.

To determine which gene modules played a key role in BPD, we used the WGCNA method to create a co-expression network and modules for both BPD and normal samples. We determined the variation of expression for each gene in GSE32472 and afterward analyzed the top 25% of genes with the greatest variance. Once the soft power value was adjusted to 19, co-expressed gene modules were determined (Figure 5A). By means of the dynamic cutting method, we obtained 14 unique co-expression modules, each of which was assigned a unique color (Figures 5B–D). Module-clinical characteristics (Control and BPD) co-expression was then continually applied to these genes in the 14 colored modules to determine adjacency and similarity. Lastly, 568 genes in the blue module showed the highest correlation with BPD (Figure 5E). In addition, we found a positive link between the blue module and module-related genes (Figure 5F).

**FIGURE 6:**
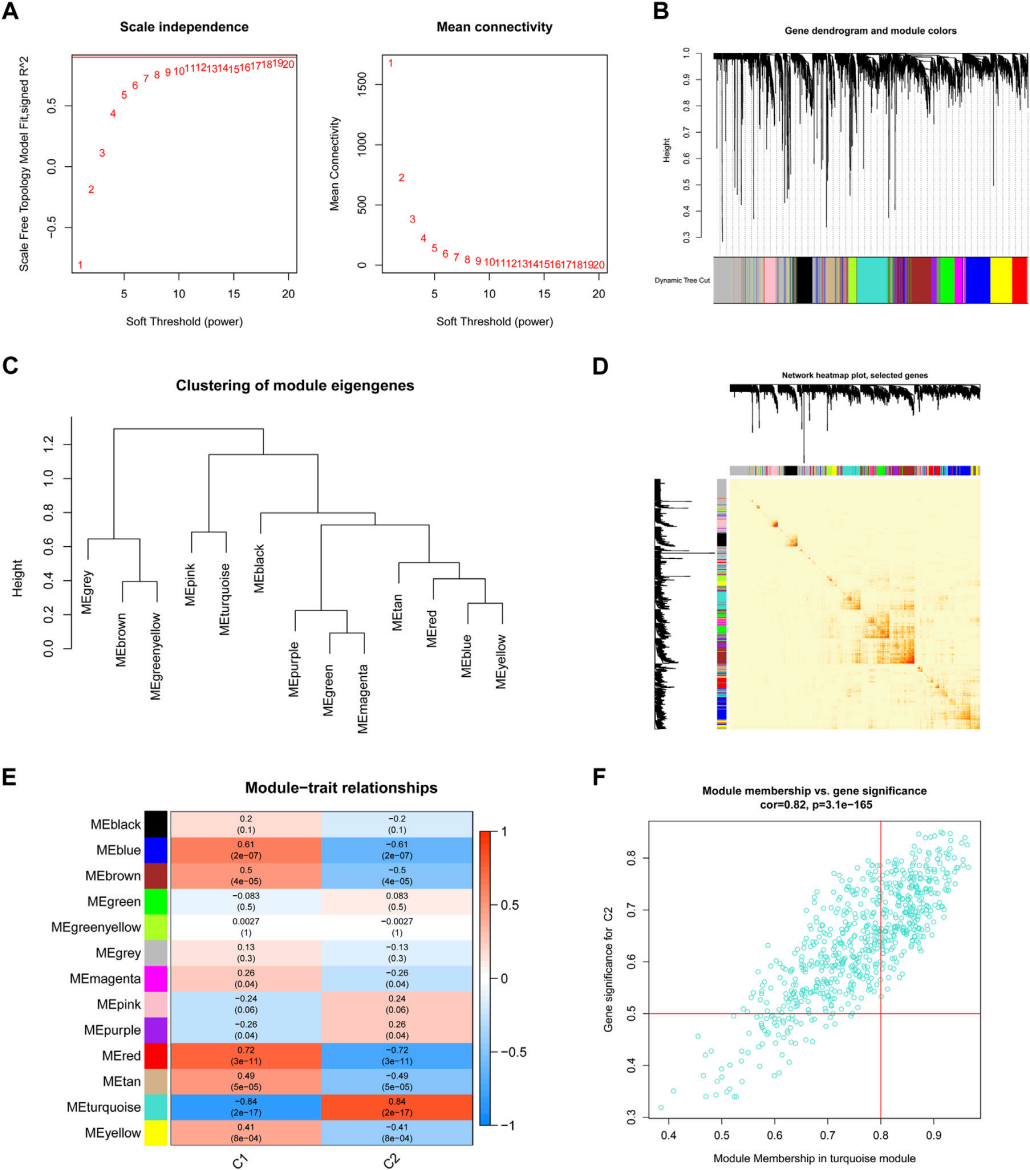
Co-expression network of DEGs between the two ER stress clusters. **(A)** The selection of soft threshold power. **(B)** Cluster tree dendrogram of co-expression modules. **(C)** Representative of clustering of module eigengenes. **(D)** Representative heatmap of the correlations among 13 modules. **(E)** Correlation analysis between module eigengenes and clinical status. **(F)** Scatter plot between module membership in turquoise module and the gene significance for cluster 2.

We used the WGCNA technique to further scrutinize the key gene modules that are linked to ER stress clusters. To develop a scale-free network, we found that a value of *β* = 19 was the optimal soft threshold (Figure 6A). In particular, a heatmap depicted the TOM of all genes associated with the 13 modules deemed to be significant (Figures 6B–D). An examination of the links between modules and clinical variables (clusters 1 and 2) revealed a strong association between the turquoise module and BPD subtypes (Figure 6E). A correlation study revealed a strong association between turquoise module genes and the chosen module (Figure 6F).

### Determination of DEGs unique to clusters and annotation of their functions

**FIGURE 7:**
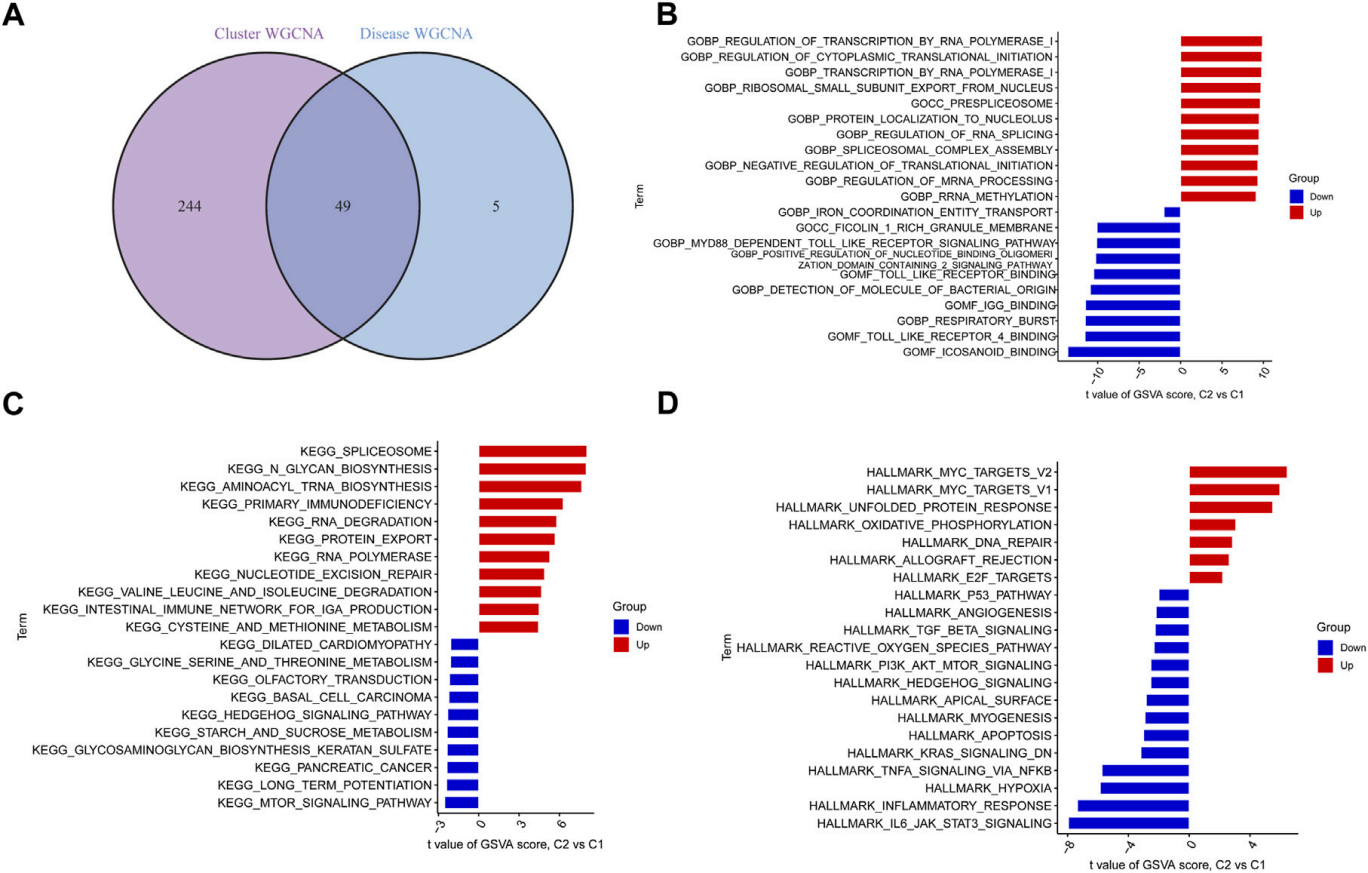
Identification of cluster-specific DEGs and biological characteristics between two ER stress clusters. **(A)** The intersections between module-related genes of ER stress clusters and module-related genes in the GSE32472 dataset. **(B–D)** Differences in GO pathway, KEGG pathway, and hallmark pathway between cluster 1 and cluster 2 samples ranked by *t*-value of GSVA method.

By intersecting the genes involved in ER stress modules with those involved in BPD and controls, we detected 49 cluster-specific DEGs (Figure 7A). To delve even further into the functional differences between the two clusters, we employed the GSVA analysis to examine the DEGs that were specific to each cluster. The findings illustrated that the regulation of transcription by RNA polymerase 1, regulation of cytoplasmic translational initiation, transcription by RNA polymerase 1, spliceosome, N glycan biosynthesis, aminoacyl tRNA biosynthesis, MYC targets, and MAPK unfolded protein response were enriched in cluster 2, while the icosanoid binding, toll-like receptor 4 binding, respiratory burst, MTOR signaling pathway, long term potentiation, IL6/JAK/STAT3 signaling, inflammatory response, and hypoxia were enriched in cluster 1 (Figures 7B–D).

### Development and evaluation of models for machine learning

**FIGURE 8:**
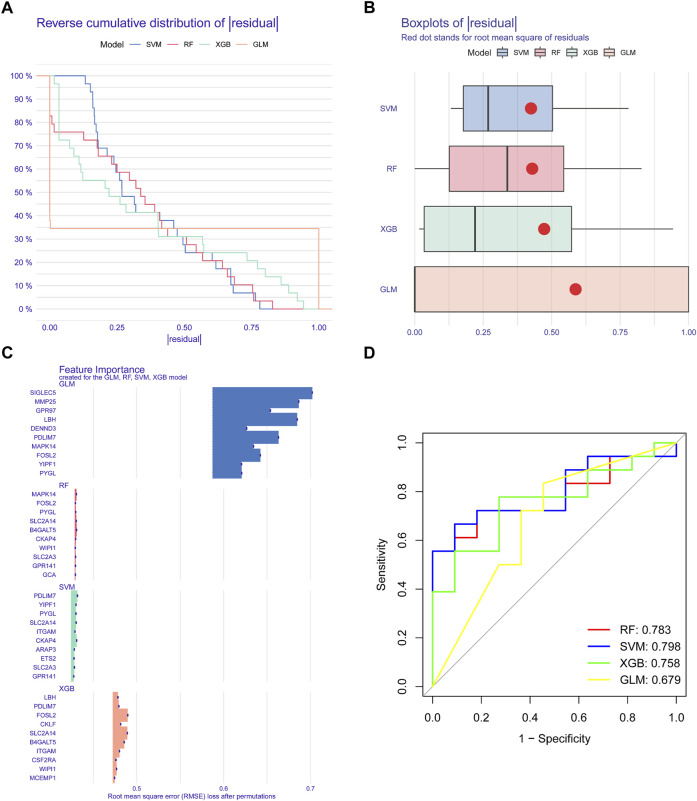
Construction and evaluation of RF, SVM, GLM, AND SGB machine models. **(A)** Cumulative residual distribution of each machine learning model. **(B)** Boxplots showed the residuals of each machine-learning model. **(C)** The important features in RF, SVM, GLM, and XGB machine models. **(D)** ROC analysis of four machine learning models based on five-fold cross-validation in the testing cohort.

We applied four validated machine learning models (SVM, RF, GLM, XGB) using the expression patterns of 49 cluster-specific DEGs in the BPD training dataset to additionally uncover subtype-specific genes with excellent diagnostic significance. The residual variance in SVM and RF machine learning models was quite low (Figures 8A, B). Next, root mean square error (RMSE) was used to determine the rank of importance for each model’s top 10 feature variables (Figure 8C). In addition, we computed ROC curves by means of 5-fold cross-validation to assess the discriminative capacity of the four machine learning techniques in the testing dataset. The area under the ROC (AUC) was greatest for the SVM machine-learning model (AUC) (RF, AUC = .783; SVM, AUC = .798; XGB, AUC = .758; GLM, AUC = .679) (Figure 8D). Altogether, these findings show that the SVM model is superior at distinguishing across patient populations. In the end, the variables PYGL, YIPF1, SLC2A14, CKAP4, and PDLIM7 that were shown to be the most significant by the SVM model were chosen to serve as predictor genes for the subsequent research.

**FIGURE 9:**
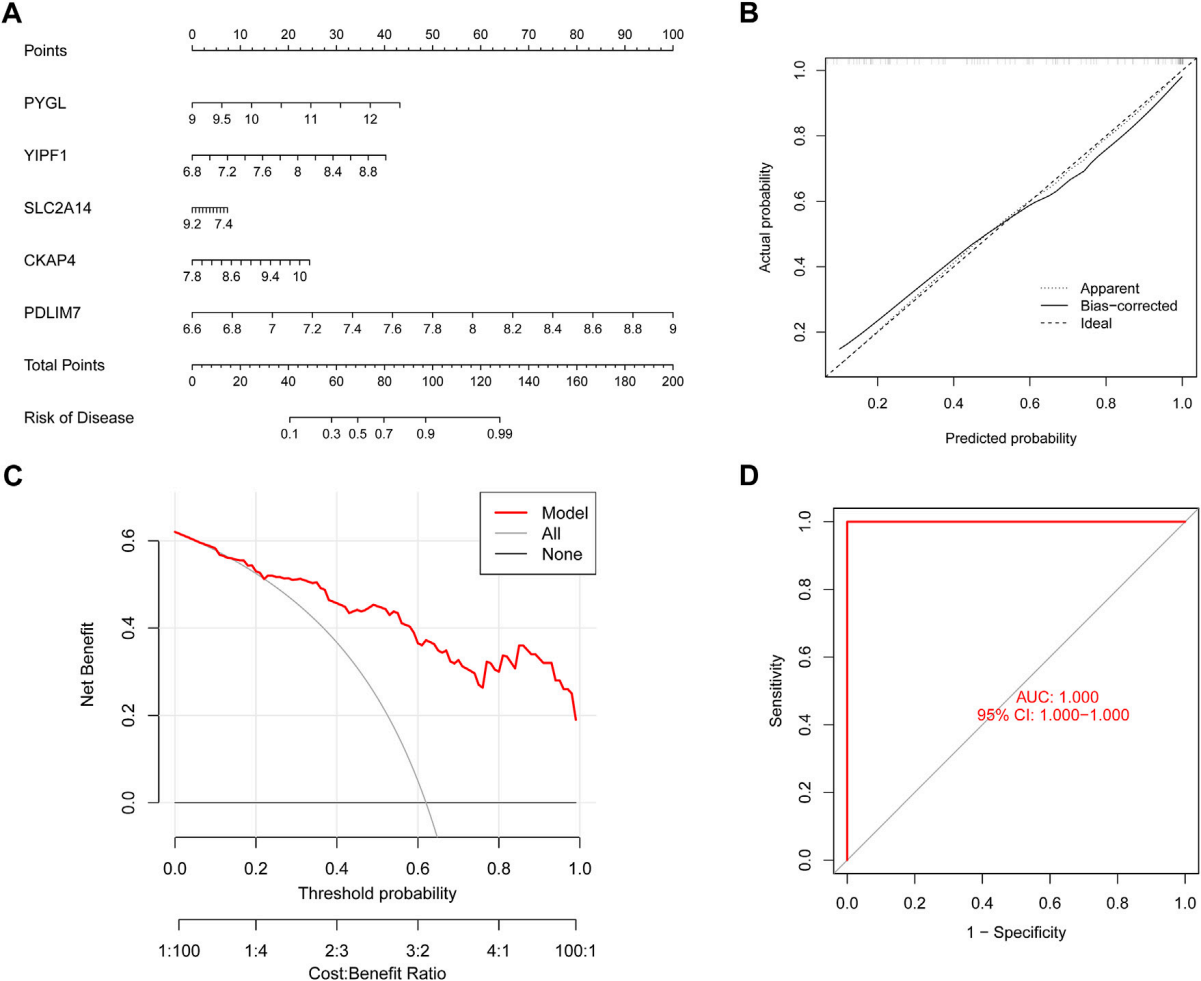
Validation of the five gene-based SVM model. **(A)** Construction of a nomogram for predicting the risk of ER stress clusters based on the five gene-based SVM model. **(B)** Calibration curve of the SVM model. **(C)** DCA curve of the SVM model. **(D)** ROC analysis of the SVM model based on five-fold cross-validation in the GSE108756 dataset.

To conduct a more in-depth analysis of the prediction power of the SVM model, we initially designed a nomogram to predict the likelihood of ER stress clusters occurring in 62 BPD patients (Figure 9A). Both the DCA and calibration curves were used to evaluate the nomogram model’s capacity for accurate prediction. The calibration curve indicated that the variation between the observed and anticipated risks of BPD clusters was low (Figure 9B), and DCA shows that our nomogram is quite accurate, highlighting that it might be used to guide medical judgment (Figure 9C). Next, we used GSE108756 to evaluate our 5-gene prognostic model, and its ROC curves demonstrated strong performance (AUC = 1.000) (Figure 9D), demonstrating that our diagnostic model is accurate in differentiating BPD cases from non-BPD individuals.

## Discussion

BPD is a complicated illness that has a significant genetic component. Some scholars contend that a variety of genetic pathways and mutations might be linked to the predisposition to developing BPD (Higano et al., 2021). Each genetic variation may participate in the development of the illness, and the accumulation of these variants results in the dysregulation of biological processes in the growing lungs of preterm babies (Jung et al., 2019). This high-risk population will benefit from the development of new treatments once the processes behind BPD are better understood. Better molecular clusters must be identified to provide direction for the customized therapy of BPD patients because of the heterogeneity of BPD pathology. The ER is the subcellular organelle that offers a one-of-a-kind environment for the synthesis of cholesterols, lipids, and proteins, as well as for the metabolism of carbohydrates and the storage of calcium (Parmar and Schroder, 2012; Sanvictores and Davis, 2022). The ER is essential to the process of alveoli production in the following ways: 1) preserving the function of the mitochondria; 2) correction of growth factor and receptor post-translation modifications; 3) reducing oxidative stress production; and 4) control of inflammation (Chan et al., 2016; Biwer and Isakson, 2017; Koksal et al., 2021). It has been observed that HOX and interferon-gamma increase ER stress in the lungs of newborn mice, thus causing lung damage and the arrival of neutrophils (Ileriturk et al., 2022). Non-etheless, further research is necessary to confirm the exact mechanisms underpinning ER stress and its modulatory involvement in different disorders. As a result, we aimed to better understand the function of ERSGs in the characterization of BPD phenotypes and the immune milieu in which they exist. Furthermore, ER stress-related gene signatures were used to classify BPD cases.

In this work, for the first time, we executed an in-depth comparison of the expression patterns of ERSGs in BPD samples with those from control groups. Compared to non-BPD patients, those with BPD were more likely to have dysregulated ERSGs, highlighting the important function of ERSGs in the development of BPD. We next determined the inter-ERSG connection to fully understand the link between ERSGs and BPD. We found that the presence of ERSG interplay in BPD patients offered evidence that some ERSGs exhibited strong synergistic or antagonistic impacts.Changes in immune cell abundance between control and BPD samples are indicative of the potential involvement of immune cells in the pathogenesis of BPD. In addition, we employed unsupervised cluster analysis to depict various ER stress trends in BPD patients according to the expression patterns of ERSGs, and we found that there were two different ER stress-related clusters. Our results found that Cluster 2 had significantly higher neutrophils. Neutrophils play a very important role in acute lung inflammation in mature and developing organisms (Beck-Schimmer et al., 2005; Phillipson and Kubes, 2011; Aggarwal et al., 2014). During the initiation of inflammation, neutrophils undergo many changes in gene expression and functional properties (Christensen, 1989). Neutrophils originate in the bone marrow and are then released into the circulatory system where they act as the first line of cellular immune defense when they are recruited to the site of injury (Furze and Rankin, 2008; Prame et al., 2018). After the first wave of inflammation, subsequent stages recruit monocytes. Neutrophils remove pathogens through phagocytosis and by releasing proteases, reactive oxygen species (ROS) and bioactive membrane vesicles (Nauseef and Borregaard, 2014). By classifying patients with BPD into Cluster 1 and Cluster 2 subtypes, we have shown that the two types of immune cells have differences, suggesting that there may be differences in outcomes that need to be explored further in the future.

Recent years have seen a surge in the use of machine learning algorithms that incorporate demographic and imaging parameters to anticipate BPD prevalence, and evidence from these research reports shows that multifactorial analyses properly accounted for links among variables, as a consequence, having a reduced error rate and more robust outcomes than univariate analysis. Premised on the expression patterns of cluster-specific DEGs, we generated an SVM-based prediction model and contrasted the prediction accuracy across four chosen machine learning classifiers (XGB, GLM, SVM, and RF), which demonstrated the best predictive efficiency in the validation cohort, revealing that SVM-based machine learning performs well in classifying BPD subtypes. Then, we built a five-gene SVM model by selecting PYGL, YIPF1, SLC2A14, CKAP4, and PDLIM7 as the most crucial variables. There is evidence that points to PYGL being involved in glycogen degradation (Zhan et al., 2021). The hypoxia metabolism gene PYGL was discovered to be upregulated in many malignancies, including breast cancers and head and neck squamous cell carcinomas (Kawakubo-Yasukochi et al., 2021; Zhao et al., 2021). The glycogen degradation mediated by PYGL has been hypothesized to sustain the growth of cancer cells. Recently, Wang et al. (2022) reported that PYGL is a potential target of miR-155-5p for regulating the function of pulmonary artery smooth muscle cells in response to hypoxia. YIPF1 belongs to the YIPF family, and HA-tagged YIPF1 has also been observed to partially localize to the ER (Soonthornsit et al., 2017). The SLC2A14 gene is responsible for encoding the glucose transporter member 14 (GLUT14). Disorders of the central nervous system, rheumatoid arthritis, lymphoma, and intraocular pressure in primary open-angle glaucoma have all been linked to mutations in the SLC2A14 gene (Amir et al., 2016; Amir et al., 2017). CKAP4 is an ER protein that is present on the surface of cell membranes and has been identified to be a cell surface receptor for many proteins such as t-PA and APF (Kimura et al., 2016; Osugi et al., 2019; Harada et al., 2020). Interaction between PDLIM7 and synaptopodin was discovered to occur through several domains, and PDLIM7 colocalizes with synaptopodin on the cisternal organelle, an unusual stacking of ER cisterns that resembles the spine apparatus, and is found at axon initial segments of a subset of neurons (D’Cruz et al., 2016).

This additional external validation in GSE108756 dataset demonstrates that the five gene-based SVM model could reliably predict BPD, expanding our understanding of the diagnostic process for BPD. Moreover, we used PYGL, YIPF1, SLC2A14, CKAP4, and PDLIM7 to develop a nomogram model for diagnosing BPD subtypes. In our tests, the model showed considerable predictive performance, suggesting its potential use in clinical settings. Altogether, the SVM model integrating these five genes provides sufficient guidance for distinguishing between BPD subtypes.

There are a few drawbacks to this research that must be mentioned. To begin, the present research relied heavily on bioinformatics, and further clinical or experimental evaluation is needed to confirm the expression patterns of ERSGs, which was not the case in our investigation. Additionally, the performance of the prediction model needs to be verified by a more comprehensive set of clinical parameters. Further investigation into the possible relationship between ERSGs and immunological responses is also warranted, and a larger number of BPD samples is required to confirm the reliability of ER stress-related clusters. Even though we used an external dataset to ensure accuracy, further trials are needed to establish a link between the characteristics of BPD pathology.

## Conclusion

Overall, our research revealed the connection between ERSGs and infiltrated immune cells and shed light on the substantial heterogeneity of the immune across BPD patients with diverse ER stress clusters. The best machine learning model for determining BPD subtypes and the clinical prognosis of BPD patients was found to be a five-gene-based SVM model. Our findings clarify the molecular processes driving BPD heterogeneity and establish for the first time the significance of ER stress in this disorder.

## Data Availability

All of the research data used in this study may be found in GEO. This database is the source for all multiple microarrays, including GSE32472 and GSE108756. R code and raw data have been uploaded to https://www.jianguoyun.com/p/DfJaN6sQk4G1CRiO4eIEIAA.
