# Supplementary material for: Identification and immunological characterization of endoplasmic reticulum stress-related molecular subtypes in bronchopulmonary dysplasia based on machine learning
Source: Front Physiol. 2023 Jan 9;13:1084650. doi: 10.3389/fphys.2022.1084650 (PMC9868568; doi:10.3389/fphys.2022.1084650)
Supplement: Supplementary file 1 [file Table2.DOCX]

<https://www.jianguoyun.com/p/DfJaN6sQk4G1CRiO4eIEIAA>
